# A case report of MoCD etiology in a neonate: A novel homozygous MoCS2 variant

**DOI:** 10.1002/ccr3.9169

**Published:** 2024-07-11

**Authors:** Jamal Sayed, Abdallah Nasir, Ahmed Gamal Sayed, Omar A. Alghamdi, Elaf Jameel Alsharif

**Affiliations:** ^1^ Neonatal Intensive Care Security Forces Hospital Makkah Makah Saudi Arabia; ^2^ College of Medicine Al‐Faisal University Riyadh Saudi Arabia; ^3^ Pediatric Department Security Forces Hospital Makkah Makkah Saudi Arabia

**Keywords:** MoCD, MoCS2, neonate, seizures

## Abstract

**Key Clinical Message:**

Molybdenum cofactor deficiency is a rare and fatal genetic disorder. Due to recurrence in the family, the etiological diagnosis could have impacted family planning and alertness to future offspring.

**Abstract:**

Molybdenum cofactor deficiency (MoCD) is a rare and fatal genetic disorder that impairs molybdenum‐dependent enzymes, resulting in conspicuous elevated urine sulfite levels and lowered serum uric acid levels. The disorder may be early‐onset, causing high fatality in neonates due to secondary complications, or late‐onset, manifesting in the first 2 years of life. Severe seizures, progressive neurological degeneration, motor abnormalities, and feeding difficulties are hallmarks of MoCD. Due to the similarity of clinical findings with those of sulfite oxidase deficiency and its neurological findings with hypoxic–ischemic encephalopathy, determining the true etiology remains challenging in MoCD patients. This case report presents a neonate in the first week of life with early onset refractory seizures, motor abnormalities, hypoactivity, and poor feeding behavior. Administering anti‐epileptic drugs did not improve the patient's condition, who started decompensating further. Nevertheless, a thorough screening for metabolic disorders revealed low serum uric acid and high sulfite levels in the urine, indicating potential MoCD. A whole exome sequencing (WES) was thus consulted for confirmatory diagnosis. Unfortunately, the patient's WES results were received after his demise, revealing MoCD caused by a novel variant of the MoCS2 gene that has not yet been reported to the best of our knowledge. This case emphasizes the need to disseminate crucial information regarding MoCD and its etiologies for prompt molecular diagnosis to reduce morbidity and mortality.

## 1 INTRODUCTION

Molybdenum cofactor deficiency (MoCD) is an extremely rare, autosomal recessive disease that results in neurometabolic disorders due to the collective impairment of molybdenum‐dependent enzymes such as sulfite and xanthine oxidases, nitrate reductases, and nitrogenases.^1^ Often misdiagnosed as hypoxic–ischemic encephalopathy (HIE), MoCD is typically characterized by refractive seizures, dystonic episodes, poor suckling, cystic encephalomalacia, neonatal encephalopathy, and mortality.^2^ Since the phenotypic spectrum of both conditions is similar, family history, and classical biochemical findings, comprising elevated S‐sulfocysteine and thiosulfate levels and decreased plasma uric acid levels, are relied upon for preliminary MoCD diagnosis.^3^ Brain magnetic resonance imaging (MRI) and electroencephalograms (EEGs) have also been used in determining brain malformations, typically observed in MoCD. However, the burst suppression patterns of neonatal MoCD patients are often misdiagnosed as epileptic or HIE.^4^ Confirmatory diagnosis is established by identifying a homozygous mutation in any of the four genes, whose protein products are responsible for molybdenum cofactor (MoCo) biosynthesis.^3^ Pathogenic variants of the molybdenum cofactor synthesis 1 (MoCS1) cause MoCD type A, those of MoCS2 cause MoCD type B, while MoCD type C is a consequence of mutations in the gephyrin (GPHN) protein.^3^ Pathogenic variants of MoCS3 have only recently been identified as an etiologic factor of MoCD.^5^

We report a male neonate who presented in the first week of life with seizures, irresponsive to anti‐epileptic drugs, and neurological assessments, corresponding to HIE. Following the patient's unfortunate demise, his whole exome sequencing (WES) reports revealed a novel MoCS2 homozygous variant responsible for neurometabolic MoCD genetic disorder. Such incidences stress the need for increased awareness regarding rare genetic disorders in neonatal intensive care, especially in neonatal encephalopathies that lead to multiple potential differential diagnoses.

## 2 CASE REPORT

### 2.1 History and examination

We describe the case of a male neonate born through cesarean section, at a gestational age of 36 weeks and 6/7 days, to first‐cousin consanguineous parents. The Apgar scores were 8 and 9 at 1 and 5 min, respectively, the baby appeared normal at birth with no unusual physical features. His anthropometric measurements were as follows: head circumference of 34.5 cm (at the 25th percentile), weight of 2.9 kg (at the 50th percentile), and length of 49 cm (at the 25th percentile), discharged home after 24 h of life in good health. On the third day, he presented to the emergency room (ER) with complaints of poor feeding behavior, and abnormal movements, such as “frequent blinking,” as described by the mother. No fever, cyanosis, history of trauma, or sick contacts were noted at the presentation. The mother was aged 28 with an obstetric history of G4P3 + A1. The family history was significant for a sibling who developed seizures and was admitted to the NICU at 48 h of life. The pregnancy was uneventful, and the baby had a history of recurrent hospital admission; his brain MRI scans revealed cortical and white matter atrophy, and the child was labeled as having cerebral palsy; he died at 5 years of age with no apparent cause of death.

During the first presentation in the ER, the infant had an episode of exaggerated rowing characterized by rapid pedaling‐like movements and tonic posturing of the upper left limb. Intense face flushing, fixed staring gaze, tachycardia of 190 beats/min, and desaturation lasting for 30 s accompanied the rowing motion, interpreted as a neonatal seizure, which ceased on administering 0.1 mg/kg lorazepam. On examination, the infant was icteric/lethargic and hypoactive, and his suck reflex was absent. Elevated lactate levels were detected by the arterial blood gas test, indicating metabolic acidosis. The patient was provisionally diagnosed with seizure disorder with metabolic acidosis, and admitted to the NICU for further follow‐up and intervention.

### 2.2 Differential diagnosis, investigations, and treatment

Following several seizures of similar semiology, and the ineffectiveness of 0.1 mg/kg lorazepam IV and 40 mg/kg levetiracetam, the neonate was electively intubated and administered an initial 0.2 mg/kg midazolam IV, followed by infusion at a 5 μg/kg/min rate. Midazolam stabilized the neonate's seizures, while intubation improved the metabolic acidosis. Initial laboratory evaluation, a full septic screen, and a comprehensive metabolic panel (CMP) were conducted, and the infant was started on IV fluids and empirical antibiotics. The CMP revealed the infant's low serum albumin level (24.00 g/L) attributable to poor feeding behavior. The most conspicuous serological findings were extremely low, almost undetectable serum uric acid levels and contrasting starkly elevated S‐sulfocysteine, xanthne, and hypoxanthine levels, as shown in Table 1.

**TABLE 1:** Molecular testing panel for determining molybdenum cofactor deficiency (MoCD).

	Levels in the patient	Reference range
Serum uric acid	0.02 mmol/L	21–42 mmol/L
S‐sulfocysteine	109 mmol/mol creatinine	<11 mmol/mol creatinine
Xanthine	213 mmol/mol creatinine	<56 mmol/mol creatinine
Hypoxanthine	87.9 mmol/mol creatinine	<65 mmol/mol creatinine

A WES was requested to confirm the suspected MoCD condition.

Simultaneously, investigations were carried out to detect any congenital defects in the patient. No intraventricular bleeding was observed in transcranial ultrasound, and glycine levels were normal in the cerebrospinal fluid (CSF). The preliminary EEG obtained from the cerebral function monitoring displayed typical status epilepticus burst‐suppression patterns and elevation attacks lasting more than 30 min.

**FIGURE 1:**
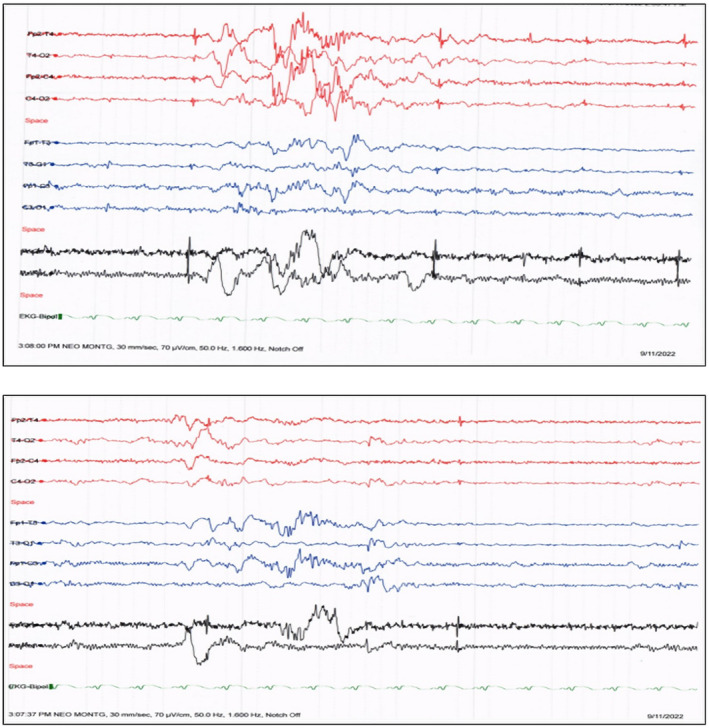
Electroencephalograms (EEG) demonstrating alternating high voltage, mixed frequency, and discontinuous background activity resembling the typical trace alternant pattern of quiet sleep in neonates. No electrographic or clinical seizure was captured during the 1‐h‐long study.

The patient's condition remained unresponsive to multiple anti‐epileptic medications despite gradually incrementing the concentrations to up to 10 mg/kg/day phenobarbitone, 60 mg/kg/day levetiracetam, and 18 μg/kg/min midazolam. The neonatal montage obtained through routine EEGs for 1 h suggested an encephalopathy state characterized by discontinuous and moderately low to medium voltage background activity; no clinical or electrographic seizures could be captured during the study (Figure 1).

**FIGURE 2:**
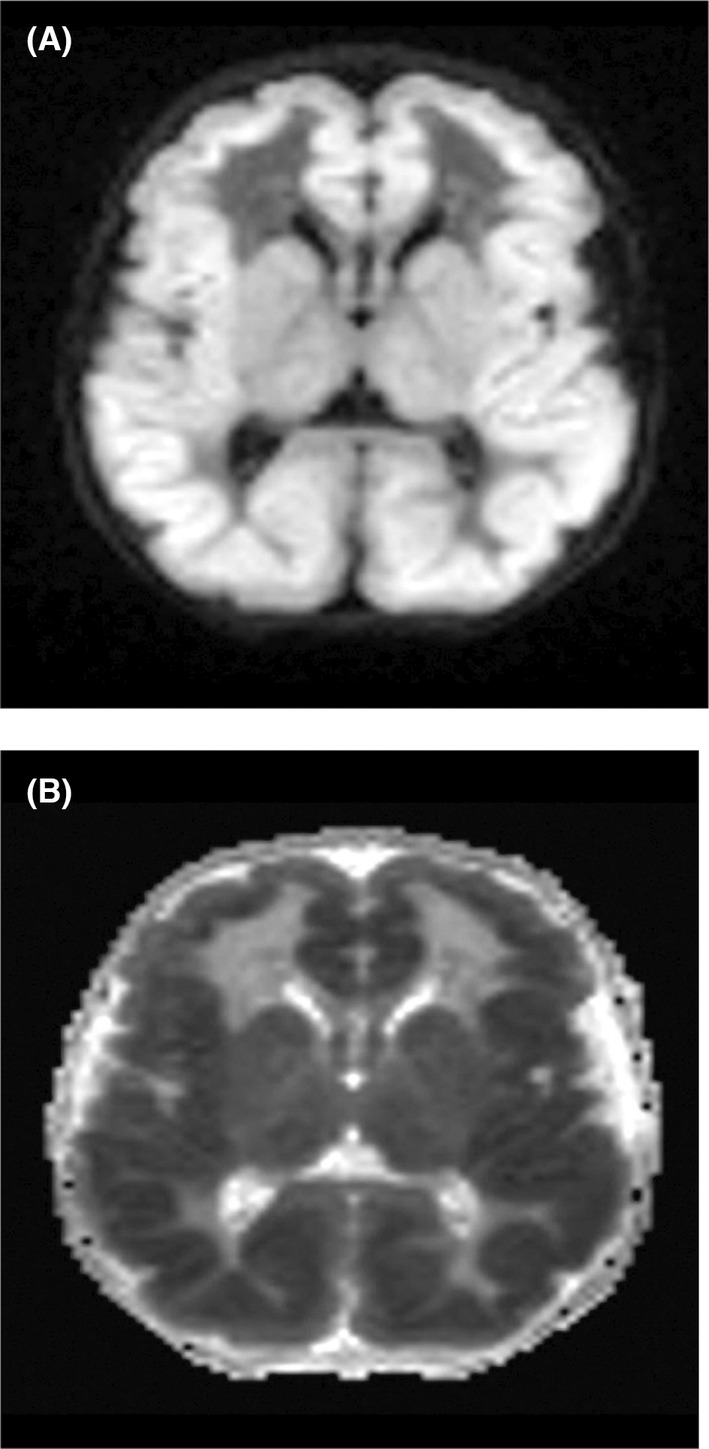
Brain MRI. Axial diffusion‐weighted imaging (DWI), (A) apparent diffusion coefficient (ADC) image. (B) Diffusion restriction (of bright signal in DWI and low signal in ADC images).

The deep sedative midazolam was weaned off following the patient's increasing bradycardia episodes, after which the neonate slowly regained consciousness but remained hypoactive despite spontaneous eye and limb activity. A magnetic resonance venogram of the cerebral veins revealed no obstruction or filling defects. An interictal EEG on the fourth day of admission resembled a trace alternate pattern, typical of quiet sleep in a neonate and could not capture any electrographic or clinical seizure during the 1‐h study. Brain MRI scans on the same day revealed diffusion restriction signals on both diffusion‐weighted images and apparent diffusion coefficient map diffusely affecting the cerebral gyri and the cerebral peduncles (Figure 2). Since no neurodegenerative etiology could be established through metabolic and radiographic analyses, we anticipated confirmation of an etiology from the WES results.

### 2.3 Outcome and follow‐up

Before the WES results came in, the infant developed edema in the limbs, which progressed into generalized edema rendering him hypotensive, with a pale and mottled look and rapid weight gain. Despite inotropic dopamine support, epinephrine infusions, and multiple resuscitation attempts, the neonate decompensated and eventually died after 24 days of life. The WES results received 6 weeks after consultation and 3 weeks after the patient's death identified a novel homozygous variant of the MOCS2 gene.

*MOCS*2 (NM_176806.4): c.30_34del (p. Leu10fs), Chr5(GRCh37): g.52404463_52404467del.

There has never been a literature report on this variant, and neither has been found in general population databases (gnomAD: no frequency). ClinVar database contains one entry for this variant with a “likely pathogenic” classification (variation ID: RCV001196957.1). This sequence change is a deletion of five nucleotides (denoted c.30_34del), which would cause a frameshift in the protein's reading frame and alter the amino acid sequence beginning at codon 10. It is expected to result in an absent or disrupted protein product. Loss‐of‐function variants in MOCS2 are known to be pathogenic (PMID: 21031595).^6^ Because there were no similar cases in the literature, it was challenging to characterize the case using VCEP criteria. Therefore, we relied on clinical features and biochemical evidence of MCOD, along with the homozygous variant's loss of function. This variant was previously reported in the ClinVar database by a single submitter as likely pathogenic.

## 3 DISCUSSION

MoCD impairs molybdenum cofactor‐dependent enzymes or molybdoenzymes, consequently affecting an array of crucial metabolic pathways. MoCD type A, caused by mutations in MoSC1, is most prevalently observed in almost 50% of reported cases.^2^ The MoCD type B, caused by MoSC2, follows closely behind and constitutes most of the remaining cases reported for MoCD. MoCD type C, caused by GPHN, is relatively rare, and MoCS3 mutations have recently been implicated in the development of MoCD.^5^, ^7^ Overlapping clinical symptoms make it challenging to differentiate the MoCD subtypes, but irrespective of gene etiology, MoCD has two distinct clinical presentations: (1) early‐onset form and (2) late‐onset form.^8^ Of the metabolic pathways affected due to MoCo deficiency, the impairment of sulfite oxidase is the most detrimental due to the accumulation of neurotoxic sulfite metabolites that cause neurometabolic degeneration of the CNS.^8^, ^9^, ^10^ Given the similarity in clinical and radiological findings of MoCD and sulfite oxidase deficiency, the accumulation of sulfites has been proposed as the major pathogenic alteration in MoCD.^10^, ^11^ The clinical, biochemical, and phenotypic spectrum of MoCD often overlaps with those of other diseases, due to which it is commonly misdiagnosed as cerebral palsy, intractable epilepsy, or HIE.^4^, ^12^, ^13^ Rarely, MoCD may result in congenital pyloric stenosis^14^ or significant xanthinuria leading to xanthine crystals or stones.^15^

The presentation of a neonate in the first week of life with seizures refractory to anti‐epileptic drugs indicates numerous differential diagnoses, which could be categorized into structural or metabolic etiologies. Cerebral MRIs can be utilized in determining brain malformations indicative of MoCD, such as cystic encephalomalacia, symmetrical pallidal or subthalamic lesions, dysgenesis of the corpus callosum, focal polymicrogyria, focal agyria, and diffuse white matter abnormalities.^8^, ^11^ Though there was no history of head trauma or prematurity, trans‐cranial ultrasound and MRIs were conducted, which tested negative for intraventricular hemorrhage, intracranial bleeding, hydrocephalus, and mass occupying lesions or ischemic changes. Early onset CNS infection is ruled out with negative blood and CSF culture. MR venography also yielded no signs of cerebral sinus thrombosis. EEGs are another valuable biomarker in diagnosing MoCD. Typical burst‐suppression background patterns, multifocal discharges in the parasagittal regions, and excess discontinuity are prevalent in the EEGs of neonatal MoCD patients.^16^ The family history of cerebral palsy in the deceased older sibling, typical status epilepticus burst suppression patterns in EEGs, and high seizure burden indicated probable congenital epilepsy of the patient. However, administration of anti‐epileptic drugs did not improve the patient's condition. Routine and extended screening for metabolic disorders yielded no positive results; WES was ordered to determine any genetic abnormalities that revealed a MoCS2 mutation, confirming MoCD type B. Exome sequencing as a first‐line diagnostic tool in this patient profile enables accurate diagnosis that could impact family planning, even in previous pregnancies.

Disease progression could be impeded in MoCD type A through cPMP injections which have shown promising results in improved seizure control, motor symptoms, and normalization of blood profiles. However, the intervention provides minimal cognitive benefit and is ineffective in MoCD type B or C.^2^ Other less efficient treatment options comprise pyridoxine supplementation for improving seizure control,^15^ dietary restriction of sulfur‐containing amino acids to reduce sulfite burden, dextromethorphan, d‐penicillamine to chelate sulfites, and anticonvulsants.^10^

## 4 CONCLUSION

Neonatal encephalopathy has multiple etiologies that extend the time required for differential diagnosis and impede proper emergency interventions. Limited awareness of rare genetic disorders further exacerbates the predicament. More studies and conventions are necessary to address such fatal genetic disorders as MoCD. Though no corrective intervention has yet been developed for any subtype of MoCD, early detection and diagnosis shall allow requisite patient management and genetic counseling for the family.

## AUTHOR CONTRIBUTIONS

**Jamal Sayed:** Conceptualization; project administration; software; writing – original draft; writing – review and editing. **Abdallah Nasir:** Writing – review and editing. **Ahmed Gamal Sayed:** Conceptualization; software; writing – review and editing. **Omar A. Alghamdi:** Conceptualization; data curation; writing – review and editing. **Elaf Jameel Alsharif:** Writing – review and editing.

## FUNDING INFORMATION

None.

## CONFLICT OF INTEREST STATEMENT

The authors declare no conflicts of interest.

## ETHICS STATEMENT

The manuscript has been reviewed and approved by the IRB and Public Affairs Office.

## CONSENT

Written informed consent was obtained from the patient to publish this report in accordance with the journal's patient consent policy.

## Data Availability

The datasets analyzed in this study are available upon request from the corresponding author. Additionally, the manuscript appropriately cites the resources used for the review and is readily accessible.
